# Self-rated health as a predictor of mortality according to cognitive impairment: findings from the Korean Longitudinal Study of Aging (2006-2016)

**DOI:** 10.4178/epih.e2021021

**Published:** 2021-04-07

**Authors:** Goun Park, Wankyo Chung

**Affiliations:** Department of Public health Science, Graduate School of Public Heath, Seoul National University, Seoul, Korea

**Keywords:** Self-rated health, Cognitive impairment, Mortality, Korea

## Abstract

**OBJECTIVES:**

Self-rated health is an instrumental variable to assess the overall health status of a population. However, it remains questionable whether it is still useful for cognitively impaired individuals. Therefore, this study aims to analyze whether self-rated health by the cognitively impaired predicts mortality reliably.

**METHODS:**

This study used 7,881 community-dwelling individuals, aged 45 and above, from the Korean Longitudinal Study of Aging (2006-2016). It used the Cox proportional hazard models for analysis. Cognitive status was classified based on the Korean Mini Mental State Examination score and a stratified analysis was used to determine whether the predictability of self-rated health varies according to cognitive status.

**RESULTS:**

For cognitively intact individuals, the adjusted hazard ratios (aHR) of mortality were 2.00 (95% confidence interval [CI], 1.18 to 3.41, model 4) for those with ‘bad’ self-rated health and 2.40 (95% CI, 1.35 to 4.25, model 4) for those with ‘very bad’ self-rated heath, respectively, compared with those with ‘very good’ health. The results remain statistically significant even after adjusting for socio-demographic factors, health status, and health-related behaviors. For cognitively impaired individuals, the aHR of mortality was statistically significant for those with ‘very bad’ self-rated health, compared with those with ‘very good’ health, when socio-demographic factors were accounted for (aHR, 3.03; 95% CI, 1.11 to 8.28, model 2).

**CONCLUSIONS:**

Self-rated health by cognitively impaired individuals remains useful in predicting mortality. It appears to be a valid and reliable health indicator for the rising population with cognitive impairment, especially caused by aging population.

## INTRODUCTION

According to the International Classification of Diseases, 11th revision, formulated by the World Health Organization, dementia is a complex syndrome resulting in reduced performance of daily activities due to reduced functions in several domains of cognitive ability (memory, execution, concentration, language, judgment, etc.) [1]. As dementia progresses, the patient loses the ability to perform daily activities, and a heavier burden of psychological and physical care is placed on the patient’s family or caregiver [2]. The number of dementia patients is rapidly increasing worldwide due to aging of the population. In 2015, the global dementia population was approximately 46.8 million, which is estimated to reach approximately 131.5 million by 2050 [2]. Likewise, in 2017 in Korea, the number of dementia patients among individuals aged ≥ 65 years was approximately 0.7 million (10.0%), which is anticipated to increase to approximately 3.02 million (16.1%) by 2050 [3,4]. The disease burden of dementia at the national level was approximately 8.7 trillion Korean won (KRW) in 2010. In addition, the per-person annual medical cost of dementia patients aged ≥ 60 years was approximately 8.05 million KRW, which is fourfold higher than that of cognitively intact elderly individuals [5]. Therefore, there is an urgent need for a valid health assessment tool and intervention for the population without the ability to take care of themselves due to cognitive impairment caused by conditions such as dementia.

The most frequently used variable for assessing the overall health status of a population is self-rated health [6]. While self-rated health is relatively easy to measure, it is a powerful and independent factor that can predict mortality and disease progression [6-10]. However, for cognitively impaired individuals such as dementia patients, the use of self-rated health as a predictor of mortality has been much disputed. This is because of the view that any information provided by these individuals is unreliable, as they show limited ability to make judgments and describe their own health status [11-13]. Thus, a commonly used method for measuring the health status of cognitively impaired individuals has been the collection of data from the caregiver regarding quality of life or depression, or the monitoring of their behaviors [12,14]. However, the report of a caregiver may differ from the actual status of the cognitively impaired individual, and a growing emphasis has been placed on the self-assessment by cognitively impaired individuals [15]. In addition, there are studies supporting the reliability of self-assessment by cognitively impaired individuals regarding the quality of life or depression, unless communication is not possible or the level of cognitive impairment is severe [12,16-18]. Nonetheless, only a few studies have investigated whether self-rated health is a reliable indicator for cognitively impaired individuals, reporting inconsistent results [19]. Walker et al. [11] showed that self-rated health could be a useful predictor of mortality in Canadian elderly individuals with mild or moderate cognitive impairment, when the participants were community residents. In contrast, Phung et al. [19] reported that mortality could not be predicted by self-rated health for early dementia patients without severe cognitive impairment. In Korea, previous studies on the predictive power of self-rated health regarding mortality have thus far reported consistent results that self-rated health is a valid predictor of mortality [20-22]. However, no study in Korea has yet investigated the same in cognitively impaired individuals [23]. The present study thus aimed to determine whether self-rated health, one of the most widely used indicators of health status, is a valid predictor of mortality in the cognitively impaired including dementia patients.

## MATERIALS AND METHODS

### Study population

This study analyzed data from the Korean Longitudinal Study of Aging (KLoSA) for the period of 2006-2016. The KLoSA provides the panel data formed based on the stratification of community residents in terms of region and residence type. A preliminary study was conducted on 10,254 residents of 6,171 houses in 2006, and a survey was performed once every two years. The KLoSA includes the data on seven categories: population, family (children, parents, siblings), health status, employment, income, asset, and subjective expectation and quality of life. The KLoSA mortality data were constructed based on the response of the family members of non-survivors in each survey since 2008. The mortality data comprise items such as the date and cause of death [24]. Of the 10,254 participants in 2006, there were 1,978 drop-outs, 163 cases of inaccurate date of death, and 232 cases of missing values, all of which were excluded from this study, which left 7,881 individuals to be included in the final analysis.

### Measures

#### Dependent variables

The survival time of non-survivors was defined as the number of days up to the date of death, as confirmed since the date of the 2006 survey. The survival time of survivors was defined as the number of days up to the date of completion of the 2016 survey. The total number of non-survivors was 1,373; 135 in 2008, 273 in 2010, 258 in 2012, 370 in 2014, and 337 in 2016.

#### Independent variables

The main explanatory variable was self-rated health, defined as the response to the question “How do you rate your health status?” among those in the 2006 survey questionnaire. The possible responses were “very good”, “good”, “fair”, “bad”, and “very bad” [6]. A five-category scale was used in this study, although previous studies on self-rated health have used both five-category and twocategory scales [22,25,26]. In addition, the Korean Mini Mental State Examination (K-MMSE) was used to differentiate the level of cognitive impairment. The K-MMSE is a tool developed by Folstein et al. [27] and translated by Kang et al. [28]. Based on the K-MMSE norms of Kang [29], which were developed in consideration of age and educational background, the K-MMSE score less than 1.5 standard deviation (SD) from the norms were considered to indicate cognitive cognitive impairment. However, as the commonly used cut-off values for cognitive impairment are ≤ 1, 1.5, or 2 SD from the norms, sensitivity analyses were carried out [30,31].

#### Control variables

The control variables were socio-demographic factors (gender, age, educational background, and marital status), health status (activities of daily living [ADLs], number of chronic diseases, disability, and depression), and health-related behaviors (smoking, problematic drinking, and regular exercise). Educational background was divided into illiterate, literate but no formal schooling, completed elementary schooling, and more than elementary schooling, based on the criteria suggested by Kang [29]. For those currently enrolled in or dropped out of elementary school, the educational background was categorized as literate but no formal schooling. Marital status was expressed as yes if currently living with a spouse and no if divorced, bereaved, seperated, or unmarried. For ADLs, the Korean Activities of Daily Living (K-ADL) developed by Katz et al. [32] and translated by Won et al. [33] for adaptation in Korea was used. Disability was defined as diagnosed by a doctor. Depression was measured using the 10-item Center for the Epidemiological Studies of Depression Short Form (CES-D-10), revised by Andersen et al. [34] by decreasing the number of questions to ten from the original tool developed by Radloff [35]. The scores for the responses in CES-D-10 were as follows: 0 for “for a brief moment or never” and “sometimes” and 1 for “frequently” and “always”[36]. A CES-D-10 score of ≥ 4 was taken to indicate the presence of depression and that of ≤ 3 was taken to indicate the absence of depression [37,38]. Individuals on anti-depressants were categorized as those with depression irrespective of participating in the CES-D-10 questionnaire. Problematic drinking was defined as two or more responses of yes on the CAGE (Cutting down, Annoyance by criticism, Guity feeling, and Eye-openers) alcohol dependence questionnaire [39]. All variables used in this study, excluding mortality and the date of death, were analyzed based on the KLoSA First Questionnaire for the period of August–December 2006.

### Statistical analysis

Table 1 shows a comparison of the characteristics of participants with different self-rated health. To analyze the intergroup differences, one-way analysis of variance was performed for continuous variables and chi-square testing was performed for categorical variables. In addition, to determine the correlation between self-rated health and mortality, the Cox proportional hazard model was used. To compare the predictive power of self-rated health according to the level of cognitive impairment, the K-MMSE was used to categorize participants as either cognitively intact or cognitively impaired for stratified analysis. Model 1 was adjusted for gender and age; model 2 was adjusted for the same variables as those adjusted in model 1, in addition to educational background and marital status; model 3 was adjusted for the same variables as those adjusted in model 2, in addition to K-ADL, number of chronic diseases, disability, and depression; model 4 was adjusted for the same variables as those of model 3, in addition to smoking, problematic drinking, and regular exercise. To analyze whether all control variables satisfied the proportionality assumption of the Cox proportional hazard model, proportionality test using Schoenfeld residuals was carried out. For all statistical analyses, Stata version 16 SE (StataCorp., College Station, TX, USA), was used.

### Ethics statement

This study was exempt from approval by the Institutional Review Board (IRB) at Seoul National University (IRB No. E1908/001-003).

## RESULTS

The results of descriptive statistics are presented in Table 1. The number of participants with self-rated health very good was 271 (3.4%), good was 2,641 (33.5%), fair was 2,518 (31.9%), bad was 1,961 (24.9%), and very bad was 490 (6.2%). The participants with very good and good self-rated health showed lower mean age and higher level of educational background. They also showed higher K-MMSE scores and lower K-ADL scores, with a lower number of chronic diseases and diagnosed cases of disability, and with more individuals also performing regular exercise.

**Figure 1.**
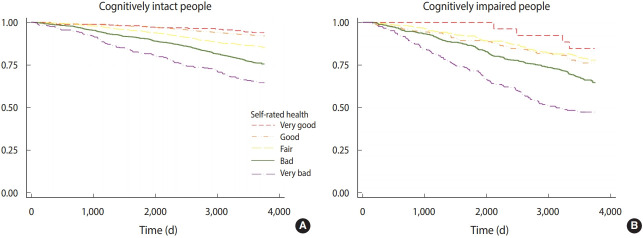
Kaplan-Meier estimates for self-rated health on mortality over ten-years (2006-2016) across different cognitive status (A) intact and (B) impaired.

Figure 1 shows a comparison of the predictive power of self-rated health using the Kaplan-Meier survival curve, where it is shown that self-rated health could predict mortality, with statistically significant differences, not only in cognitively-intact but also in cognitively impaired individuals.

Table 2 presents the results of stratified analysis for the correlation between self-rated health and mortality using the Cox proportional hazard model, according to the level of cognitive impairment. For cognitively intact individuals, the hazard ratio (HR) was significantly higher for those with bad or very bad self-rated health than for those who responded very good (self-rated health, bad: model 1: HR, 2.33; 95% confidence interval [CI], 1.38 to 3.93; model 2: HR, 2.33; 95% CI, 1.38 to 3.93; model 3: HR, 2.05; 95% CI, 1.21 to 3.49; and model 4: HR, 2.00; 95% CI, 1.18 to 3.41; very bad: model 1: HR, 3.46; 95% CI, 2.00 to 5.97; model 2: HR, 3.28; 95% CI, 1.89 to 5.68; model 3: HR, 2.50; 95% CI, 1.41 to 4.43; and model 4: HR, 2.40; 95% CI, 1.35 to 4.25). Statistical significance was maintained from models 1 to 4. The same results were obtained when the analysis was carried out for all participants. Compared to individuals with very good self-rated health, the HR was significantly higher for those who responded bad or very bad for self-rated health (self-rated health, bad: model 1: HR, 2.25; 95% CI, 1.42 to 3.56; model 2: HR, 2.24; 95% CI, 1.41 to 3.56; model 3: HR, 1.98; 95% CI, 1.24 to 3.16; and model 4: HR, 1.94; 95% CI, 1.22 to 3.10; very bad: model 1: HR, 3.58; 95% CI, 2.23 to 5.75; model 2: HR, 3.46; 95% CI, 2.15 to 5.57; model 3: HR, 2.53; 95% CI, 1.54 to 4.15; and model 4: HR, 2.41; 95% CI, 1.47 to 3.95). For cognitively impaired individuais, however, the HR was significantly higher for those with very bad self-rated health than for those who responded very good, while statistical significance was shown to be maintained only up to model 2, where gender, age, marital status, and educational background were controlled (selfrated health, very bad: model 1: HR, 2.94; 95% CI, 1.08 to 8.00 and model 2: HR, 3.03; 95% CI, 1.11 to 8.28).

## DISCUSSION

This study verified whether the predictive power of self-rated health varied according to cognitive impairment, in line with the currently increasing number of cognitively impaired individuals due to aging. The results showed that, for cognitively intact individuals and for all participants, the HR of those who responded bad or very bad for self-rated health was significantly higher than that for those with very good self-rated health, regardless of the model used. For cognitively impaired individuals, the difference between the participants with very good self-rated health and those with very bad self-rated health was statistically significant in the models adjusted only for socio-demographic factors.

Our results were in partial agreement with those from a study conducted by Walker et al. [11], which was conducted with community-dwelling participants like this study. In the study conducted by Walker et al. [11], the predictive power of self-rated health was shown to decrease with the fall in cognitive ability. For cognitively intact individuals, the HR based on self-rated health was 1.57 (95% CI, 1.38 to 1.78), while that for individuals with mild or moderate cognitive impairment was 1.26 (95% CI, 1.01 to 1.59). For individuals with severe cognitive impairment, however, self-rated health did not predict mortality. Thus, except in the case of severely reduced cognitive ability, the study provided evidence to support the idea that self-rated health was a valid predictor of mortality. The fact that self-rated health did not predict mortality in individuals with severe cognitive impairment was attributed to the fall in cognitive ability, accompanied by the reduced ability to integrate the necessary data for self-rated health [11].

The results were in disagreement with those of another previous study on Alzheimer’s patients without severe cognitive impairment (Mini Mental State Examination [MMSE]≥ 20) that reported self-rated health as being unsuitable as a predictor of mortality. Phung et al. [19] examined patients with Alzheimer’s disease, analyzing both the patient-rated health and caregiver-rated health with respect to the correlation with mortality. The results showed that, although patient-rated health did not predict mortality, caregiver-rated health could predict mortality, with a statistically significant difference. They also showed that early dementia patients tended to rate their health higher than the caregivers. Likewise, Nielsen et al. [40] showed that mortality could not be predicted by self-rated health in patients with mild Alzheimer’s disease without severely reduced MMSE. As the MMSE score and self-awareness of disease decreased, the probability that the patient would rate their health as high increased. Waldorff et al. [41] also showed that, as the MMSE score and self-awareness of disease decreased in early Alzheimer’s patients, the self-rated health was high. The results collectively indicated that the patients differed from cognitively intact individuals in assessing health.

The differences among previous studies may be attributed to the fact that, while the participants in the three studies[19,40,41] were patients diagnosed with Alzheimer’s disease, participants in Walker et al. [11] and those in the present study were community residents. What this implies is that, for community residents, self-rated health adequately accounts for the overall health status of a given community. Thus, even in the current situation wherein the aged population and cognitively impaired individuals are increasing in proportion, self-rated health can be used as an indicator of the overall health status of a community.

In future, the rate of increase in the number of dementia patients and cognitively impaired individuals is expected to be higher due to rapid aging. In line with this, approximately 17% of the participants in this study showed reduced cognitive abilities, with a substantial number of participants showing a low K-MMSE score. Therefore, the level of cognitive impairment should be considered when using self-rated health as an actual substitute for health indicators in aged or cognitively impaired individuals. Moreover, in this study, self-rated health by cognitively impaired individuals was shown to be a valid predictor of mortality in models 1 and 2. Above all, as self-rated health is a unique expression of one’s own health by an individual, self-rated health by cognitively impaired individuals should always be respected. In addition, as the ability to recognize and analyze the disease would decline in cognitively impaired individuals, an integrative health assessment tool for these individuals should be developed to allow continuous monitoring, assessment, and intervention of their health.

This study is significant in that it verified self-rated health as a valid predictor of mortality using the national data of KLoSA, while considering the level of cognitive impairment for the first time. The level of pain, quality of life, and depression in cognitively impaired individuals had been continuously studied, and most studies on this topic have reported that the self-rated measures should be valued [12,15,17,18]. However, only a few studies have reported on the self-rated health of cognitively impaired individuals and none on the predictive power regarding mortality in Korea. With the currently increasing number of cognitively impaired individuals in each community due to aging, the findings in this study are anticipated to prove useful in the health assessment of the aged population.

The limitations in this study should be acknowledged. First, the reliability of self-rated health as an indicator was examined based solely on mortality among various health indices. Self-rated health is a factor related not only to disease but also to socioeconomic status and psychological health in complexity [42]. Thus, the relationship between self-rated health and mortality may be affected by other variables which are uncontrolled in this study. Second, the data of inaccurate date of death or missing values were excluded from analysis in this study, and it is possible that the resulting selectivity bias had an influence on the results. Thus, the descriptive statistics of the group including the data and of the group excluding the data are presented for comparison in Supplementary Material 1. As shown, the two groups did not vary significantly in the distribution of K-MMSE, chronic disease, disability, problematic drinking, or regular exercise, indicating that the influence of selection bias on the results was negligible. Third, since previous studies suggested different criteria for categorizing self-rated health, additional analyses were carried out using a two-category scale, constructed based on the five-category scale used in this study; the results are presented in Supplementary Materials 2 and 3. When “fair” in the five-category scale was merged as “good” in the two-category scale, self-rated health could predict the mortality of cognitively impaired individuals, with a statistically significant difference, but when it was merged as “bad” in the two-category scale, the mortality could not be predicted with statistical significance. Fourth, the cut-off of the K-MMSE for differentiating the level of cognitive impairment has continuously been disputed. In this study, the norms of Kang [29], who developed the K-MMSE, were used. Nonetheless, as different studies suggested different cut-offs for K-MMSE, a sensitivity analysis was carried out by recategorizing cognitively impaired individuals using KMMSE scores ≤ 1 or 2 SD [30,31]. The results are presented in Supplementary Materials 4 and 5. The results of the sensitivity analysis showed that statistical significance and coefficient size did not differ significantly between ≤ 1.5 SD and ≤ 1 or 2 SD as the cut-off. Lastly, the presence of cognitive impairment in this study was based on the K-MMSE scores, while the study participants were residents of a community. Further studies should thus investigate whether self-rated health is a valid predictor of mortality in patients actually diagnosed with dementia and in the elderly at a sanatorium or care facility.

## Figures and Tables

**Table 1.** Distribution of sample characteristics at baseline, 2006

Characteristics		Self-rated health					Total (n=7,881)	p-value
		Very good (n=271)	Good (n=2,641)	Fair (n=2,518)	Bad (n=1,961)	Very bad (n=490)		
Gender								<0.001
	Men	164 (60.5)	1,397 (52.9)	1,102 (43.8)	643 (32.8)	167 (34.1)	3,473 (44.1)	
	Women	107 (39.5)	1,244 (47.1)	1,416 (56.2)	1,318 (67.2)	323 (65.9)	4,408 (55.9)	
Age		55.96±9.60	57.43±9.47	62.25±10.45	66.64±9.94	68.58±9.98	61.91±10.73	<0.001
Education								<0.001
	Illiterate	8 (2.9)	54 (2.0)	105 (4.2)	250 (12.7)	119 (24.3)	536 (6.8)	
	Literate but no formal schooling	4 (1.5)	167 (6.3)	366 (14.5)	493 (25.1)	113 (23.1)	1,143 (14.5)	
	Completed elementary	33 (12.2)	489 (18.5)	738 (29.3)	628 (32.0)	145 (29.6)	2,033 (25.8)	
	More than elementary education	226 (83.4)	1,931 (73.1)	1,309 (52.0)	590 (30.1)	113 (23.1)	4,169 (52.9)	
Marriage								<0.001
	Yes	245 (90.4)	2,332 (88.3)	2,023 (80.3)	1,360 (69.3)	307 (62.6)	6,267 (79.5)	
	No	26 (9.6)	309 (11.7)	495 (19.7)	60 1(30.6)	183 (37.3)	1,614 (20.5)	
K-MMSE		27.38±3.90	27.47±3.42	25.86±4.37	23.05±5.81	20.24±7.38	25.04±5.19	<0.001
	Cognitively intact	245 (90.4)	2,465 (93.3)	2,157 (85.7)	1,415 (72.2)	287 (58.6)	6,569 (83.3)	<0.001
	Cognitively impaired	26 (9.6)	176 (6.7)	361 (14.3)	546 (27.8)	203 (41.4)	1,312 (16.6)	
K-ADL		7.01±0.09	7.02±0.41	7.04±0.43	7.22±1.20	8.29±3.05	7.16±1.07	<0.001
No. of chronic diseases		0.26±0.54	0.31±0.5)	0.67±0.80	1.28±1.04	1.78±1.28	0.76±0.95	<0.001
Disabled								<0.001
	Yes	0 (0.0)	55 (2.1)	102 (4.0)	228 (11.6)	115 (23.5)	500 (6.3)	
	No	271 (100)	2,586 (97.9)	2,416 (95.9)	1,733 (88.4)	375 (76.5)	7,381 (93.7)	
Depression								<0.001
	Yes	7 (2.6)	34 (1.3)	141 (5.6)	419 (21.4)	271 (55.3)	872 (11.1)	
	No	264 (97.4)	2,607 (98.7)	2,377 (94.4)	1,542 (78.6)	219 (44.7)	7,009 (88.9)	<0.001
CES-D-10		1.35±1.19	1.23±1.04	1.55±1.32	2.41±2.19	4.50±2.93	1.83±1.83	
Smoking status								<0.001
	Non-smoker	184 (67.9)	1,772 (67.1)	1,778 (70.6)	1,501 (76.5)	337 (68.8)	5,572 (70.7)	
	Former smoker	31 (11.4)	237 (9.0)	262 (10.4)	192 (9.8)	63 (12.9)	785 (10.0)	
	Current smoker	56 (20.7)	632 (23.9)	478 (19.0)	268 (13.7)	90 (18.4)	1,524 (19.3)	
Problematic drinking								0.640
	Yes	6 (2.2)	76 (2.9)	80 (3.2)	48 (2.4)	14 (2.9)	224 (2.8)	
	No	265 (97.8)	2,565 (97.1)	2,438 (96.8)	1,913 (97.5)	476 (97.1)	7,657 (97.2)	
Regular exercise								<0.001
	Yes	155 (57.2)	1,181 (44.7)	932 (37.0)	608 (31.0)	107 (21.8)	2,983 (37.8)	
	No	116 (42.8)	1,460 (55.3)	1,586 (63.0)	1,353 (69.0)	383 (78.2)	4,898 (62.1)	

Values are presented as number (%) or mean±standard deviation. K-MMSE, Korean Mini Mental State Examination; K-ADL, Korean Activities of Daily Living; CES-D-10, 10-item Center for the Epidemiological Studies of Depression Short Form.

**Table 2.** Hazard ratios of self-rated health across different cognitive status

Self-rated health	Cognitively intact (n=6,569)	Cognitively impaired (n=1,312)	Total (n=7,881)
Model 1			
Very good	1.00 (reference)	1.00 (reference)	1.00 (reference)
Good	1.21 (0.72, 2.05)	1.67 (0.60, 4.66)	1.23 (0.77, 1.96)
Fair	1.54 (0.92, 2.60)	1.24 (0.46, 3.40)	1.46 (0.92, 2.32)
Bad	2.33 (1.38, 3.93)^**^	1.82 (0.67, 4.89)	2.25 (1.42, 3.56)^**^
Very bad	3.46 (2.00, 5.97)^***^	2.94 (1.08, 8.00)^*^	3.58 (2.23, 5.75)^***^
Model 2			
Very good	1.00 (reference)	1.00 (reference)	1.00 (reference)
Good	1.22 (0.72, 2.06)	1.71 (0.61, 4.80)	1.24 (0.78, 1.98)
Fair	1.57 (0.93, 2.64)	1.27 (0.46, 3.49)	1.48 (0.93, 2.35)
Bad	2.33 (1.38, 3.93)^**^	1.87 (0.69, 5.07)	2.24 (1.41, 3.56)^**^
Very bad	3.28 (1.89, 5.68)^***^	3.03 (1.11, 8.28)^*^	3.46 (2.15, 5.57)^***^
Model 3			
Very good	1.00 (reference)	1.00 (reference)	1.00 (reference)
Good	1.20 (0.71, 2.03)	1.61 (0.57, 4.53)	1.22 (0.77, 1.95)
Fair	1.50 (0.89, 2.52)	1.19 (0.43, 3.27)	1.42 (0.89, 2.25)
Bad	2.05 (1.21, 3.49)^**^	1.60 (0.58, 4.37)	1.98 (1.24, 3.16)^**^
Very bad	2.50 (1.41, 4.43)^**^	2.14 (0.76, 6.01)	2.53 (1.54, 4.15)^***^
Model 4			
Very good	1.00 (reference)	1.00 (reference)	1.00 (reference)
Good	1.18 (0.70, 1.99)	1.57 (0.56, 4.41)	1.21 (0.76, 1.93)
Fair	1.46 (0.87, 2.47)	1.13 (0.41, 3.12)	1.39 (0.87, 2.21)
Bad	2.00 (1.18, 3.41)^*^	1.59 (0.58, 4.36)	1.94 (1.22, 3.10)^**^
Very bad	2.40 (1.35, 4.25)^**^	2.03 (0.72, 5.73)	2.41 (1.47, 3.95)^**^

Values are presented as hazard ratio (95% confidence interval). Model 1: adjusted for gender and age; Model 2: adjusted for the variables included in model 1 and additionally those for education and marriage; Model 3: adjusted for the variables included in model 2 and additionally those for the Korean Activities of Daily Living, number of chronic disease, being disabled and having depression; Model 4: adjusted for the variables included in model 3 and additionally those for smoking status, problematic drinking and regular exercise.

* p<0.05,

** p<0.01,

*** p<0.001.

## SUPPLEMENTARY MATERIALS

Korean version is available at http://www.e-epih.org/.

Supplementary Material 1. Comparison of sample characteristics at baseline between included and excluded individuals from the analysis(Year=2006)

Supplementary Material 2. Sensitivity analysis: Hazard Ratios of Poor self-rated health between (Very Good, Good, Fair) and (Bad, Very Bad)

Supplementary Material 3. Sensitivity analysis: Hazard Ratios of Poor self-rated health between (Very Good, Good) and (Fair, Bad, Very Bad)

Supplementary Material 4. Sensitivity analysis: Hazard Ratios of Poor self-rated health using a different cutoff point(1SD)

Supplementary Material 5. Sensitivity analysis: Hazard Ratios of Poor self-rated health using a different cutoff point(2SD)
