# Supplementary material for: Self-rated health as a predictor of mortality according to cognitive impairment: findings from the Korean Longitudinal Study of Aging (2006-2016)
Source: Epidemiol Health. 2021 Apr 7;43:e2021021. doi: 10.4178/epih.e2021021 (PMC8289473; doi:10.4178/epih.e2021021)
Supplement: Supplementary file 1 [file epih-43-e2021021-suppl1.docx]

**Title** : (국문) 인지 저하 수준에 따른 주관적 건강의 사망예측력 변화

(영문) Self-rated health as a predictor of mortality according to cognitive impairment

**Full names of all authors** : Goun Park, Wankyo Chung

**ORICD** : (Goun Park) 0000-0002-6471-5270

(Wankyo Chung) 0000-0001-8094-2433

**Affilations** : Graduate School of Public Heath, Seoul National University

**Corresponding author:** Wankyo Chung, Ph.D. in Health Economics,

Professor, Graduate School of Public Health & SNU AI Institute (AIIS)

Seoul National University

(08826) 1, Gwanak-ro, Gwanak-gu, Seoul, Republic of Korea

Graduate School of Public Heath

E-mail : wankyo@snu.ac.kr

**Running title** : (국문)주관적 건강의 사망예측력 변화

(영문) Self-rated health and mortality

**Abstract**

[Background]

주관적 건강은 인구 집단의 전반적인 건강 상태를 사정하는 대표적인 변수로 쓰인다. 그러나, 인지 저하자들에게도 주관적 건강이 그들의 건강 상태를 나타내는 변수인지는 의문이 있다. 따라서, 본 연구는 인지 저하자들이 평가한 주관적 건강이 사망을 예측할 수 있는지 분석하고자 한다.

[Methods]

본 연구는 2006년부터 2016년까지의 고령화연구패널조사(KLoSA)자료를 이용하여 45세 이상 지역사회 거주자 7,881명을 대상으로 분석을 시행하였다. 분석 방법은 콕스 비례 위험 모델(Cox proportional Hazard Model)을 사용하였다. 인지 저하에 따라 주관적 건강의 사망예측력이 달라지는지 확인하기 위해, K-MMSE 점수에 의거 인지 저하 여부를 구분하여 층화 분석을 시행하였다.

[Results]

정상 인지군에서, 주관적 건강을 매우 좋다고 평가한 집단을 기준으로 했을 때, 주관적 건강을 나쁘다, 매우 나쁘다고 평가한 집단의 사망위험비가 각각 2배(Model4 HR:2.00,95%CI:1.18-3.41), 2.40배(Model4 HR:2.40,95%CI:1.35-4.25)로 확인되었다. 사회인구학적 요인, 건강 상태 및 건강 관련 습관 변수들을 다양하게 통제한 모델 1부터 4까지 통계적 유의성이 유지되었다. 인지 저하군에서는 주관적 건강을 매우 좋다고 평가한 집단을 기준으로 했을 때, 매우 나쁘다고 평가한 집단의 사망위험비가 사회인구학적 요인을 통제한 모델 2까지 통계적으로 유의하게 높았다(Model2 HR:3.03,95%CI:1.11-8.28).

[Conclusions]

인지 저하군이 평가한 주관적 건강은 사망을 통계적으로 유의하게 예측하였다. 인구의 고령화로 지역 사회 내에서 인지 저하자가 지속적으로 증가하고 있는 상황에서 주관적 건강은 유효한 사망예측 인자이며 신뢰할 만한 건강지표이다.

**key words**

Self-rated health, Cognitive impairment, Mortality, Korea

**Main text**

1. Background

세계보건기구의 국제질병분류(ICD-11: International Classification of Disease)에 따르면, 치매는 인지의 여러 영역들(기억력, 실행 능력, 집중력, 언어, 판단력 등)의 기능저하로 일상생활 수행 능력이 감소되는 복합 증후군이다 [1]. 치매 환자는 치매가 진행됨에 따라 일상생활을 유지하기가 어려워져 환자의 가족이나 돌봄 제공자에게 정신적, 육체적인 돌봄 부담을 가중시키게 된다 [2]. 전 세계적으로 고령화가 진행되면서 치매 환자 또한 급속도로 증가하고 있다. 2015년 기준 전 세계의 치매 인구는 약 4,680만 명이며, 이는 2050년까지 약 1억 3150 만 명에 이를 것으로 추정된다 [2]. 한국의 경우에도, 2017년 65세 이상 인구 중 치매 환자는 약 70만 명(10%)으로 추산되며, 이는 2050년에 약 302만 명(16.09%)으로 증가할 것으로 예상된다 [3, 4]. 2010년 기준 국가 전체의 치매로 인한 질병 부담은, 8조 7천억원에 육박하는 수준이었다. 또한, 60세 이상 치매 환자의 일인당 연간 의료비는 805만 원으로, 정상 노인에 비해 4배가량 의료비 지출이 높은 것으로 확인되었다 [5]. 따라서, 치매 등의 인지 저하로 자신의 건강을 관리하지 못하는 인구 집단을 위한 건강 사정과 정책적 개입이 매우 필요한 실정이다.

인구 집단을 대상으로 전체적인 건강 상태를 측정할 때, 가장 많이 쓰이는 변수 중 하나는 주관적 건강이다 [6]. 주관적 건강은 각 개인이 자신의 건강을 평가한 것으로 측정하기 쉬우면서도, 사망과 질병 경과를 예측할 수 있는 강력하고 독립적인 변수이다 [6-10]. 그러나 치매환자와 같은 인지 저하자의 경우, 주관적 건강이 사망을 예측할수 있는 변수인지에 대해서 논란이 있다. 인지 저하자는 자신의 상태에 대해 판단하고 표현하는 능력이 제한되어, 인지 저하자가 제공한 정보를 신뢰하기 어렵다는 견해가 있기 때문이다 [11-13]. 따라서 인지 저하자의 삶의 질이나, 우울감 등의 상태를 측정할 때는 돌봄 제공자에게서 관련 정보를 제공받거나, 인지저하자의 행동을 통해 상태를 측정하는 방법이 사용되어 왔다 [12, 14]. 하지만, 돌봄 제공자의 보고는 인지 저하자의 실제 상태와 다를 수 있기 때문에, 인지 저하자가 자신의 상태를 직접적으로 표현하는 것의 중요성이 커지고 있다 [15]. 또한, 의사소통이 불가하거나 인지 저하가 매우 심하지 않은 이상, 인지 저하자의 삶의 질, 우울감 등에 관한 인지 저하자의 자기 표현이 신뢰할 만하다는 연구 결과도 존재한다 [12, 16-18]. 그러나, 인지 저하자의 주관적 건강이 신뢰할 만한 지표인가를 밝힌 연구는 매우 드물며, 그 결과도 연구마다 각각 다르게 제시한다 [19]. Walker et al.(2004)는 캐나다의 지역사회 거주 노인을 대상으로 한 연구에서, 경도 및 중등도 인지 저하자들에게서도 주관적 건강이 사망의 유용한 예측 인자가 될 수 있음을 밝혔다 [11]. 하지만 Phung et al.(2018)은 인지 저하가 심하지 않은 초기 치매 환자들이 평가한 주관적 건강이 사망을 예측하지 못한다는 결과를 제시하였다 [19]. 주관적 건강의 사망예측력을 주제로 한 국내 연구들은 주관적 건강이 사망을 예측할 수 있다는 일관된 결과를 제시하고 있다 [20-22]. 그러나, 한국에서 인지저하자를 대상으로 한 주관적 건강의 사망 예측력 연구는 전무하다 [23]. 따라서 본 연구는 인구 집단의 건강 상태를 사정하기 위해 가장 널리 쓰이는 지표 중의 하나인 주관적 건강이 치매환자를 비롯한 인지 저하자들에게도 사망에 대한 유효한 예측 변수인지를 분석하고자 한다.

2. Methods

1) Study population

본 연구는 2006년부터 2016년까지의 고령화연구패널조사(Korean Longitudinal Study of Aging)와 고령화연구패널조사 사망자 자료를 분석자료로 이용하였다. 고령화연구패널조사는 45세 이상의 지역사회 거주자들을 지역 및 주거형태별로 층화하여 구축한 패널 자료이다. 2006년 6,171가구에 거주하는 10,254명을 대상으로 기본 조사가 이루어졌으며, 2년마다 1회의 설문조사를 시행하였다. 고령화연구패널조사는 인구, 가족(자녀, 부모, 형제자매), 건강상태, 고용, 소득, 자산, 주관적 기대감 및 삶의 질 등의 7가지 항목으로 구성되어 있다. 고령화연구패널조사 사망자 자료는 2008년부터 매 설문조사시마다 사망자 발생 가구구성원의 응답을 기초로 만들어졌다. 사망자 자료는 사망일시, 사망원인 등의 항목이 조사되어 있다 [24]. 본 연구에서는 2006년 10,254명의 표본 중 중도탈락자 1978명, 사망 날짜가 부정확한 경우 163명, 결측치 232명을 제외하였다. 최종 분석에 사용된 표본은 7881명이다.

2) Ethics Statement

이 연구는 서울대학교 생명윤리위원회로부터 심의 면제 대상임을 인정받았다(IRB No. E1908/001-003).

3) Measures

Dependent variable

사망자의 생존 기간은 2006년 설문조사 시작일로부터 확인된 사망일까지의 일 수로, 생존자의 생존 기간은 2006년 설문조사 시작일로부터 2016년 설문조사 종료일까지의 일 수로 정의하였다. 사망자의 사망일 확인에 사용된 자료는 2008년부터 2016년까지의 고령화연구패널조사 사망자 자료이며, 총 사망자 1373명은 2008년에 135명, 2010년에 273명, 2012년에 258명, 2014년에 370명, 2016년에 337명이 조사되었다.

Independent variable

주요 설명변수인 주관적 건강은 2006년 설문조사 질문 중 하나인 “본인의 건강상태에 대해 어떻게 생각하십니까?”에 대한 응답으로 정의하였으며, 그 결과를 매우 좋음, 좋음, 보통, 나쁨, 매우 나쁨으로 분류하였다 [6]. 주관적 건강과 관련된 선행 연구들이 주관적 건강을 오분척도로 뿐만 아니라 이분척도로도 제시하나, 본 연구에서는 오분척도를 사용하여 분석을 시행하였다[22, 25, 26].

또한, 인지 저하 수준을 구분하기 위해, Folstein et al.(1975)가 제작하고, 강연욱 등(1997)이 번안한 K-MMSE(Korean Mini Mental State Exam, 이하 K-MMSE)를 사용하였다 [27, 28]. K-MMSE 점수를 강연욱(2006)의 규준에 의거하여 연령, 교육 수준에 따른 절단점 변화를 고려, K-MMSE 점수가 규준에서 1.5 표준편차 아래 있을 때 인지 저하군으로 분류하였다 [29]. 그러나, 인지 저하군을 분류하는 절단점은 평균에서 1 표준편차 이하, 1.5 표준편차 이하, 2 표준편차 이하가 모두 흔히 쓰이기에, 민감도 분석을 또한 시행하였다[30, 31].

Control variables

통제 변수로는 사회인구학적 요인(성별, 연령, 교육 수준, 결혼 여부), 건강 상태(ADL 점수, 만성질환 개수, 장애 여부, 우울 여부), 건강 관련 습관(흡연 여부, 문제 음주 여부, 운동 여부) 등이 있다. 교육 수준은 강연욱(2006) 이 제시한 규준에 따라 무학, 무학이나 문맹 아님(0-3년), 초등학교 졸업, 초등학교 졸업 이상으로 분류하였다 [29]. 교육 수준이 초등학교 중퇴 및 재학인 경우 무학이나 문맹 아님으로 분류하였다. 결혼 여부는 배우자가 현재 있을 경우에만 '예'로, 이혼, 사별, 이산가족 및 미혼일 경우에는 '아니오'로 나누었다. 일상생활수행능력(Activities of Daily Living, 이하 ADL) 점수로는 Katz et al.(1963)이 개발하고 원장원 등(2002)이 한국의 상황에 맞게 번안한 K-ADL(Korean Activities of Daily Living, 이하 K-ADL)를 사용하였다 [32, 33]. 장애 여부는, 의사로부터 장애 판정을 받았을 시 장애가 있다고 분류하였다. 우울 여부를 판단하는 도구는 Radloff(1977)이 제작한 CESD 문항을 Andersen et al(1994) 가 10문항으로 단축한 CESD-10 도구를 사용하였다 [34, 35]. CESD-10 질문에 대한 응답 중 '1. 잠깐 그런 생각이 들었거나 그런 생각이 들지 않았음.' 과 '2. 가끔 그런 생각이 들었음'을 0점으로, '3. 자주 그런 생각이 들었음' 과 '4. 항상 그런 생각이 들었음'을 1점으로 하여 질문의 점수를 합산하였다 [36]. CESD-10 점수가 4점 이상시 우울감 있음, 3점 이하시에는 우울감 없음으로 분류하였다 [37, 38]. 본 연구에서 항우울제를 복용하고 있는 사람들은 CESD-10문항에 참여하지 않아, 우울감 있음으로 분류하였다. 문제 음주 여부는 CAGE 음주 의존도 문항에서 2개 이상을 “예”라고 응답하였을 때, 문제 음주로 정의하였다 [39]. 본 연구에 사용된 사망 여부와 사망일시를 제외한 모든 변수들은 2006년 8월부터 12월까지 시행된 고령화연구패널조사 1차 설문조사 자료를 이용하였다.

4) Statistical analysis

표 1에서는 주관적 건강을 서로 다르게 평가한 연구대상자의 특성을 비교하였다. 연속 변수의 경우에는 one way anova test를, 범주형 변수인 경우에는 Chi-square test를 이용하여 집단간의 차이를 확인하였다. 또한, 주관적 건강과 사망간의 연관성을 파악하기 위해 콕스 비례위험 모델(Cox Proportional Hazard Model)을 사용하였다. 인지 저하 수준에 따른 주관적 건강의 사망 예측력을 비교하기 위해서, K-MMSE 점수에 의거하여 인지 저하 수준을 정상, 인지저하군으로 나누어 층화 분석을 시행하였다. 모델 1에서 성별과 연령을, 모델 2 에서 모델 1 변수 외에 교육 수준과 결혼 여부를, 모델 3에서 모델 2 변수 외에 K-ADL 점수, 만성 질환 개수, 우울 여부, 장애 여부를, 모델 4에서 모델 3 변수 외에 흡연 여부, 문제 음주 여부, 운동 여부를 각각 통제하였다. 모든 통제 변수가 콕스 비례 위험 모델의 비례성 가정을 충족시키는지 확인하기 위해 Schoenfeld 잔차를 이용한 비례성 검증을 시행하였다. 통계 분석 패키지는 STATA version 16 SE (StataCorp., College Station, TX, USA)를 사용하였다.

3. Results

기술통계량 분석 결과는 Table1에 제시되었다. 주관적 건강이 매우 좋다고 평가한 사람은 271명(3.4%), 좋다고 평가한 사람은 2641명(33.5%), 보통은 2518명(31.9%), 나쁨은 1961명(24.9%), 매우 나쁨은 490명(6.2%)이었다. 주관적 건강이 좋다고 평가한 집단일수록 평균연령이 낮았으며, 교육 수준이 높은 경향이 있었다. 주관적 건강이 좋다고 평가한 집단은 K-MMSE 점수가 더 높았으며, K-ADL 점수는 더 낮았다. 주관적 건강을 좋다고 평가할수록 만성 질환의 개수, 장애 판정을 받은 경우가 적었다. 주관적 건강이 좋다고 평가한 집단은 규칙적인 운동을 하는 경우가 많았다. [table 1 삽입]

Figure 1에서는 주관적 건강의 사망예측력을 Kaplan-Meier 생존곡선으로 비교하였으며, 정상 인지군에서 뿐만 아니라 인지 저하군에서도 주관적 건강이 사망을 유의하게 예측하는 것이 확인되었다[Figure 1 삽입]. Table 2에서는 주관적 건강과 사망과의 관련성을 콕스 비례위험 모델을 이용하여 인지 저하 수준에 따라 층화분석한 결과를 제시하였다. 분석 결과, 정상 인지군에서는 주관적 건강이 매우 좋다고 대답한 그룹을 기준으로 했을 때, 주관적 건강이 나쁘다, 매우 나쁘다고 대답한 그룹의 사망위험비가 통계적으로 유의하게 높았다(Self-rated health bad : Model 1 HR=2.33(95%CI:1.38-3.93), Model2 HR=2.33(95%CI:1.38-3.93), Model3 HR=2.05(95%CI:1.21-3.49), Model 4 HR=2.00(95%CI:1.18-3.41), Self-rated health very bad : Model 1 HR=3.46(95%CI:2.00-5.97), Model2 HR=3.28(95%CI:1.89-5.68), Model3 HR=2.50(95%CI:1.41-4.43), Model4 HR=2.40(95%CI:1.35-4.25)). 통계적 유의성은 Model 1 에서 Model 4 까지 유지되었다. 전체 집단을 대상으로 했을 때에도 정상 인지군과 동일한 결과가 확인되었다. 주관적 건강이 매우 좋다고 대답한 그룹 대비, 나쁘다 및 매우 나쁘다고 대답한 그룹의 사망위험비가 통계적으로 유의하게 높았다(Self-rated health bad : Model 1 HR=2.25(95%CI:1.42-3.56), Model2 HR=2.24(95%CI:1.41-3.56), Model3 HR=1.98(95%CI:1.24-3.16), Model 4 HR=1.94(95%CI:1.22-3.10), Self-rated health very bad : Model 1 HR=3.58(95%CI:2.23-5.75), Model2 HR=3.46(95%CI:2.15-5.57), Model3 HR=2.53(95%CI:1.54-4.15), Model4 HR=2.41(95%CI:1.47-3.95)). 하지만, 인지 저하군에는, 주관적 건강을 매우 좋다고 대답한 그룹에 비해 매우 나쁘다고 대답한 그룹만 사망위험비가 통계적으로 높았으며, 성별, 연령, 결혼 여부, 교육수준을 통제한 Model 2 까지만 통계적 유의성이 유지되었다(Self-rated health very bad Model 1 HR=2.94(95%CI:1.08-8.00), Model2 HR=3.03(95%CI:1.11-8.28)). [table2 삽입]

4. Discussion

본 연구는 고령화로 인해 인지 저하자가 증가하는 현 상황에서, 인지 저하 정도에 따라 주관적 건강의 사망예측력이 달라지는지 분석하였다. 분석결과를 살펴보면, 정상 인지군과 전체 집단에서는 사용된 다양한 모델과 상관없이 주관적 건강이 '매우 좋음'으로 대답한 집단을 기준으로 했을 때, 나쁨, 매우 나쁨으로 대답한 집단의 사망위험비가 통계적으로 유의하게 높았다. 인지 저하군에서는 '매우 좋음' 대비 '매우 나쁨' 집단간의 차이가 인구사회학적 요인만 통제했을 경우 통계적으로 유의하였다.

이러한 결과는 본 연구와 같이 지역사회 인구집단을 대상으로 한 Walker et al.(2004)의 연구와 부분적으로 일치하는 것이다 [11]. Walker et al.(2004)는 인지 능력이 저하됨에 따라 주관적 건강의 사망예측력이 감소되는 결과를 제시하였다. 정상 인지군의 경우 주관적 건강의 사망위험비가 1.57배(95% CI:1.38-1.78), 경도와 중등도 인지 저하자의 경우 1.26배(95% CI:1.01-1.59)로 확인되었으며, 중증 인지 저하자의 경우에는 주관적 건강이 사망을 예측하지 못하였다. 따라서, 인지 능력이 매우 저하되는 경우를 제외하면, 주관적 건강이 사망을 예측하는 유효한 변수라는 근거를 제시하였다. 그리고 중증 인지 저하자의 주관적 건강이 사망을 예측하지 못하는 것은, 인지 능력이 저하될수록 주관적 건강평가시 필요한 정보를 통합하는 능력도 저하되기 때문이라고 설명하였다 [11].

이러한 결과는 인지 저하가 심하지 않은(MMSE≥20) 알츠하이머 환자들에게서, 주관적 건강이 사망의 예측 변수가 되지 못한다는 내용의 다른 선행 연구와는 차이가 있다. Phung et al.(2018)은 알츠하이머 환자의 주관적 건강과 보호자가 평가한 환자의 건강을 구분하여 각각 사망과의 연관성을 분석하였다. 분석에 따르면, 알츠하이머 환자의 주관적 건강은 사망을 예측하지 못하였으나, 보호자가 평가한 환자의 건강은 사망을 유의하게 예측하였다. 또한, Phung et al.(2018)은 초기 치매 환자들이 자신의 건강을 보호자보다 더 좋게 평가하는 경향이 있었음을 보였다 [19]. Nielsen et al.(2016)도 MMSE 저하가 심하지 않은 경증 알츠하이머 환자들이 평가한 주관적 건강이 사망을 예측할 수 없다는 결과를 제시하였다. MMSE 점수가 낮아지고, 자신의 질병에 대한 인식이 저하될수록 주관적 건강을 좋게 평가할 가능성이 크다는 것을 확인하였다 [40]. Waldorff et el.(2010)에서도, 초기 알츠하이머 환자들이 MMSE 점수가 저하될수록, 질병에 대한 인식이 없을수록 주관적 건강을 좋게 평가하여, 인지저하가 없는 사람들과 주관적 건강 평가를 달리 한다고 결론지었다 [41].

연구 결과들 간의 이러한 차이는 앞의 세 연구(Phung et al.(2018), Nielsen et al.(2016), Waldorff et al.(2010))의 연구 대상자가 모두 알츠하이머 진단을 받은 환자인 반면, Walker et al.(2004)와 본 연구는 지역사회 인구 집단을 대상으로 하였기 때문으로 판단된다 [19, 40, 41]. 이는 지역사회 인구집단을 대상으로 할 경우, 주관적 건강이 인구 집단의 건강 상태를 충분히 설명할 수 있다는 것을 의미한다. 따라서 노인 인구와 인지 저하자들이 증가하는 현 상황에서도 주관적 건강은 지역사회 인구 집단의 건강 상태를 측정하는 지표로 사용 가능하다는 것이다.

향후, 급격한 고령화에 따라 치매 환자와 인지 저하자의 증가는 가속화될 것으로 예상된다. 본 연구에서도, 연구대상자의 약 17%가 인지 저하군으로 상당히 많은 수의 인구가 K-MMSE 점수 저하가 있는 것으로 확인되었다. 따라서 고령인구와 인지 저하자를 대상으로 주관적 건강을 실제 건강의 대리 변수로 활용하게 될 때에는 인지 저하 정도를 중요하게 고려하여야 할 것이다. 또한, 인지 저하자의 주관적 건강은 모델 1,2에서 사망을 예측할 수 있다고 확인되었으며, 무엇보다도 개인의 건강에 대한 고유한 표현이기에, 인지저하자의 주관적 건강 표현은 존중되어야 할 것이다. 더불어, 인지 저하자는 점차 자신의 질병을 인식하고 판단하는 능력이 저하되기에, 지속적인 건강 사정과 중재를 할 수 있는 종합적인 인지저하자 건강 사정 도구가 필요함을 제언한다.

이 연구는 국내 자료를 통해서는 처음으로 인지 저하 수준을 고려하여 주관적 건강이 유효한 사망예측 인자임을 분석하였다는 점에서 의의가 있다. 인지 저하자의 통증, 삶의 질, 또는 우울과 관련해서는 국내에서 지속적인 연구가 있었고 대개 인지 저하자의 자기 표현이 존중되어야 한다는 결과들을 제시하였다 [12, 15, 17, 18]. 하지만, 인지 저하자의 주관적 건강에 대한 연구는 극히 드물었으며, 그 사망예측력에 관한 연구는 없었다. 인구의 고령화로 지역 사회 내에서 인지 저하자가 지속적으로 증가하고 있는 상황에서 이러한 연구 결과는 고령 인구 집단의 건강 사정에 보탬이 될 수 있을 것으로 기대한다.

본 연구의 한계점은 첫째, 주관적 건강의 신뢰성을 여러 건강 지표 가운데 사망으로만 확인하였다는 것이다. 주관적 건강은 질병뿐만 아니라, 사회경제적 수준, 정신적 건강 등과 복합적으로 관련이 있는 변수이다 [42]. 따라서, 주관적 건강과 사망의 연관성이 본 연구에서 통제되지 않은 다른 변수들에 의해 다르게 나타날 수 있다. 둘째, 사망 날짜가 정확하지 않을 경우 및 자료의 결측치가 있을 경우 분석 대상에서 제외하였으므로 선택 편의가 결과에 영향을 미쳤을 수 있다. 따라서, 분석 포함 그룹과 미포함 그룹의 기술통계량을 Supplement table 1 에 비교 제시하였다. 그 결과 분석 포함 그룹과 미포함 그룹은 K-MMSE 점수, 만성 질환, 장애 여부, 문제 음주, 운동 여부 등의 분포에 있어 통계적으로 유의한 차이가 없다고 확인되어, 선택 편의에 의한 결과의 차이는 크지 않을 것이라 판단된다. 셋째, 주관적 건강의 분류 기준에 대해서도 연구자마다 각기 다른 기준을 제시하고 있어, 주관적 건강의 오분척도를 이분척도로 재분류 하여 그 결과를 Supplement table 2-3 에 제시하였다. 주관적 건강이 오분척도의 ‘보통’인 경우 그 보통을 ‘좋음’ 이분척도에 포함하였을 때는 인지 저하자의 주관적 건강이 사망을 유의하게 예측할 수 있었으나, '보통'을 '나쁨' 이분척도에 포함시켰을 때는 인지 저하자의 주관적 건강이 사망을 유의하게 예측하지 못하였다. 넷째, 인지 저하 수준을 구분하는 K-MMSE의 절단 기준과 관련하여 많은 논란이 있다. 본 연구에서는 K-MMSE를 번안한 강연욱(2006)의 규준을 사용하였다 [29]. 그러나 K-MMSE 절단 기준은 연구마다 각각 다르게 제시하고 있으므로 본 연구는 강연욱(2006)의 규준을 기준으로 1 표준편차 이하 및 2 표준편차 이하의 K-MMSE 점수를 이용하여 인지 저하군을 재분류하여 민감도 분석을 시행하였고, 그 결과를 Supplement table 4-5 에 제시하였다. 1 표준편차 이하 및 2 표준편차 이하의 절단 기준을 사용하여 민감도 분석을 시행하였을 때, 1.5 표준편차 이하의 절단 기준을 사용하였을 때 통계적 유의성과 계수 크기에서 큰 차이가 없았다. 마지막으로, 본 연구는 지역사회집단을 대상으로 하여 K-MMSE 점수를 기준으로 인지 저하 여부를 판단한 연구이다. 따라서, 실제 치매진단을 받은 환자가 대상일 때, 또한 요양원 및 시설입소 노인을 대상으로 할 때 주관적 건강의 사망예측력이 유효한지에 대한 후속 연구를 제안한다.

5.Reference

1. World Health Organization. ICD-11 for Mortality and Morbidity Statistics;2020[cited 2020.Jul 8].Available from : <https://icd.who.int/browse11/l-m/en#/http://id.who.int/icd/entity/546689346>

2.Prince M, Wimo A, Guerchet M, Ali MG, Wu YT, Prina M. World Alzheimer report 2015: the global impact of dementia-an analysis of prevalence, incidence, cost and trends.[cited 2020 Jul 8]. Available from:<https://www.alz.co.uk/research/WorldAlzheimerReport2015.pdf>.

3. National Institute of Dementia. Korean dementia observatory 2018 [cited 2020 Jul 8]. Available from: https://www.nid.or.kr/info/dataroom_view.aspx?bid= 194 (Korean).

4. National Institute of Dementia. Nationalwide survey on the dementia epidemiology of Korea 2016 [cited 2020 Jul 8]. Available from: https://www.nid.or.kr/info/dataroom_view.aspx?bid= 182(Korean).

5.Kim KU, Kwak KP, Kim KS, Kim MD, Kim BJ, Kim SK, et al. Survey of dementia in elderly. Seoul: Ministry of Health and Welfare; 2011, p. 20-351 (Korean, authors translation)

6.Jylhä M. What is self-rated health and why does it predict mortality? Towards a unified conceptual model. Social Science & Medicine 2009;69:307-316.

7.Fayers PM, Sprangers MAG. Understanding self-rated health. The Lancet 2002;359:187-188.

8.DeSalvo KB, Bloser N, Reynolds K, He J, Muntner P. Mortality Prediction with a Single General Self-Rated Health Question. Journal of General Internal Medicine 2006;21:267-275.

9.Idler EL, Benyamini Y. Self-Rated Health and Mortality: A Review of Twenty-Seven Community Studies. Journal of Health and Social Behavior 1997;38:21-37.

10.Kaplan GA, Camacho T. PERCEIVED HEALTH AND MORTALITY: A NINE-YEAR FOLLOW-UP OF THE HUMAN POPULATION LABORATORY COHORT1. American Journal of Epidemiology 1983;117:292-304.

11.Walker JD, Maxwell CJ, Hogan DB, Ebly EM. Does Self-Rated Health Predict Survival in Older Persons with Cognitive Impairment? the American Geriatric Society 2004;52:1895-1900.

12.Shin KR, Kang YH, Jung DY, Kim RH, Whang SN. Comparison between Perception of Dementia Patients’ and Caregivers’ Assessment on Patients' Anxiety, Depression, and Quality of Life. Korean Journal of Adult Nursing 2008;20:804-814.

13.Kim HS, Yu SJ. Pain Assessment in Nonverbal Older Adults with Dementia. Korean journal of hospice and palliative care 2013;16:145-154.

14.Albert SM, Del Castillo-Castaneda C, Sano M, Jacobs DM, Marder K, Bell K, et al. Quality of life in patients with Alzheimer's disease as reported by patient proxies. Journal of American Geriatrics Society 1996;44:1342-1347.

15.Shin HY. A Preliminary Study on Korean Version of the Quality of Life-Alzheimer’s Disease (QOL-AD) in Community-Dwelling Elderly with Dementia. Journal of Preventive Medicine and Public Health 2006;39:243-248.

16.Logsdon RG, Gibbons LE, McCurry SM, Teri L. Assessing quality of life in older adults with cognitive impairment. Psychosomatic Medicine 2002;64:510-519.

17.Park BH, Lee TJ, Lee YS, Jang SH, Choi NH, Jeong HG, et al. Cost of Illness and Quality of Life of Patients and Their Caregivers with Mild Cognitive Impairment or Alzheimer’s Disease. Journal of Health Tech Assess 2019;7:62-74.

18.Ryu YS, Park JS. Development and Effect of Evidence-based Nursing Practice Guidelines for Pain Management in Patients with Dementia. Korean J Adult Nurs 2019;31:176-189.

19.Phung TKT, Siersma V, Vogel A, Waldorff FB, Waldemar G. Self-rated versus Caregiver-rated Health for Patients with Mild Dementia as Predictors of Patient Mortality. American Journal of Geriatric Psychiatry 2018;26:375-385.

20.Oh HC, Jee SH, Kim IS. Self Rated Health and Mortality in Elderly Kangwha Cohort, 8-year follow up. Epidemiol Health 1994;16:172-180.

21.Woo HK, Moon OR. The difference Mortality According to Self-Assessed Health Status Korean J. of Health Policy & Administration 2008;18:49-65.

22.Khang YH, Kim HR. Self-rated health and mortality: gender- and age-specific contributions of explanatory factors in South Korea. International Journal of Public Health 2010;55:279-289.

23.Oh SK, Lee TR. Comparison of Cognitive Function, Barthel Activities of Daily Living and Self-Perceived Health Toward Elders and Demented Elders. Journal of The Korea Society of Health Informatics and Statistics 2011;36:1-13.

24.Korea Employment Information Service;2006[cited 2020 Jul 2]. Available from: <https://survey.keis.or.kr/klosa/klosa03.jsp>

25.Yoon BJ. Differential Effects on Self-rated Health by Socioeconomic Class. Journal of Health Informatics and Statistics 2016;41:35-42.

26.Kim HR. The Relationship of Socioeconomic Position and Health Behaviors with Morbidity in Seoul, Korea. Health and Social Welfare Review 2005;25:3-35.

27.Folstein MF, Folstein SE, McHugh PR. “Mini-mental state”: a practical method for grading the cognitive state of patients for the clinician. Journal of Psychiatric Research 1975;12:189-198.

28.Kang YW, Na DL, Hahn SH. A validity Study on the Korean Mini-Mental State Examination(K-MMSE) in Dementia Patients Journal of the Korean Neurological Association 1997;15:300-308.

29.Kang YW. A Normative Study of the Korean-Mini Mental State Examination(K-MMSE) in the Elderly The Korean Journal of the psychology : General 2006;25:1-12.

30.Han C, Jo SA, Jo I, Kim E, Park MH, Kang Y. An adaptation of the Korean mini-mental state examination (K-MMSE) in elderly Koreans: Demographic influence and population-based norms (the AGE study). Archives of Gerontology and Geriatrics 2008;47:302-310.

31.Feinstein AR. Clinical epidemiology: the architecture of clinical research. Philadelphia: WB Saunders; 1985, p. 671.

32.Katz S, Ford AB, Moskowitz RW, Jackson BA, Jaffe MW. Studies of Illness in the Aged: The Index of ADL: A Standardized Measure of Biological and Psychosocial Function. JAMA 1963;185:914-919.

33.Won CW, Yang KY, Rho YG, KIm SY, Lee E, Yoon JL, et al. The development of Korean activities of Daily Living(K-ADL) and Korean Instrumental Activities of Daily Living(K-IADL) Scale Annals of geriatric medicine and research 2002;6:pp.107-120.

34.Andresen EM, Malmgren JA, Carter WB, Patrick DL. Screening for Depression in Well Older Adults: Evaluation of a Short Form of the CES-D. American Journal of Preventive Medicine 1994;10:77-84.

35.Radloff LS. The CES-D Scale:A Self-Report Depression Scale for Research in the General Population. Applied Psychological Measurement 1977;1:385-401.

36.Kohout FJ, Berkman LF, Evans DA, Cornoni-Huntley J. Two Shorter Forms of the CES-D Depression Symptoms Index. Journal of Aging and Health 1993;5:179-193.

37.Cheng ST, Chan ACM. The Center for Epidemiologic Studies Depression Scale in older Chinese: thresholds for long and short forms. International Journal of Geriatric Psychiatry 2005;20:465-470.

38.Bae SW, Kim, Yi-Young, Doh, Moonhak, Kim, Hyeji, Park, Byung-Sun. Testing factor structure and measurement invariance of 10-item versions of the CES-D scale : Focusing on Andersen form and Boston form of the CES-D-10. Mental Health & Social Work 2020;48:33-55.

39.Ewing JA. Detecting Alcoholism: The CAGE Questionnaire. JAMA 1984;252:1905-1907.

40.Nielsen ABS, Siersma V, Waldemar G, Waldorff FB. Poor self-rated health did not increase risk of permanent nursing placement or mortality in people with mild Alzheimer’s disease. J BMC Geriatrics 2016;16:8.

41.Waldorff FB, Nielsen ABS, Waldemar G. Self-rated health in patients with mild Alzheimer's disease: baseline data from the Danish Alzheimer Intervention Study. Archives of Gerontology and Geriatrics 2010;50:1-5.

42.Singh-Manoux A, Martikainen P, Ferrie J, Zins M, Marmot M, Goldberg M. What does self rated health measure? Results from the British Whitehall II and French Gazel cohort studies. International Journal of Epidemiology 2006;60:364-372.
