# Supplementary material for: Self-rated health as a predictor of mortality according to cognitive impairment: findings from the Korean Longitudinal Study of Aging (2006-2016)
Source: Epidemiol Health. 2021 Apr 7;43:e2021021. doi: 10.4178/epih.e2021021 (PMC8289473; doi:10.4178/epih.e2021021)
Supplement: Supplementary Material 1. — Comparison of sample characteristics at baseline between included and excluded individuals from the analysis(Year=2006) [file epih-43-e2021021-suppl2.docx]

| **Supplementary Material 1. Comparison of sample characteristics at baseline between included and excluded individuals from the analysis(Year=2006)** | | | | |
| --- | --- | --- | --- | --- |
| **Characteristics** | **Included individuals**  **(n=7881)** | **Excluded individuals**  **(n=2373)** | **Total**  **(n=10254)** | **P-value** |
| **Self-rated health** |  |  |  |  |
| **Very Good** | 271(3.44) | 86(3.62) | 357(3.48) | <0.001 |
| **Good** | 2641(33.51) | 861(36.28) | 3502(34.15) |  |
| **Fair** | 2518(31.95) | 690(29.08) | 3208(31.29) |  |
| **Bad** | 1961(24.88) | 526(22.17) | 2487(24.25) |  |
| **Very Bad** | 490(6.22) | 210(8.85) | 700(6.83) |  |
| **Death** |  |  |  |  |
| **Died during follow up** | 1373(17.42) | 285(12.01) | 1658(16.17) | <0.001 |
| **Alive during follow up** | 6508(82.58) | 2088(87.99) | 8596(83.83) |  |
| **Gender** |  |  |  |  |
| Male, n(%) | 3473(44.07) | 990(41.72) | 4463(43.52) | 0.043 |
| Female, n(%) | 4408(55.93) | 1383(58.28) | 5791(56.48) |  |
| **Age** |  |  |  |  |
| Mean(SD) | 61.91(10.73) | 61.04(12.32) | 61.71(11.12) | <0.001 |
| **Education** |  |  |  |  |
| Illiterate, n(%) | 536(6.80) | 173(7.29) | 709(6.91) | <0.001 |
| Literate but no formal schooling, n(%) | 1143((14.50) | 293(12.35) | 1436(14.00) |  |
| Completed elementary, n(%) | 2033(25.80) | 471(19.85) | 2504(24.42) |  |
| More than elementary education, n(%) | 4169(52.90) | 1436(60.51) | 5605(54.66) |  |
| **Marriage** |  |  |  |  |
| Yes, n(%) | 6267(79.52) | 1704(71.81) | 7971(77.74) | <0.001 |
| No, n(%) | 1614(20.48) | 669(28.19) | 2283(22.26) |  |
| **K-MMSE(1.5SD)** |  |  |  |  |
| Cognitively intact | 6569(83.35) | 1800(83.33) | 8369(83.35) | 0.98 |
| Cognitively impaired | 1312(16.65) | 360(16.67) | 1672(16.65) |  |
| Mean(SD) | 25.40(5.19) | 25.48(5.85) | 25.42(5.34) | 0.56 |
| **K-ADL, Mean(SD)** | 7.16(1.07) | 7.58(2.43) | 7.25(1.51) | <0.001 |
| **Number of chronic diseases, Mean(SD)** | 0.76(0.95) | 0.74(0.97) | 0.75(0.96) | 0.38 |
| **Disabled** |  |  |  |  |
| Yes, n(%) | 500(6.34) | 169(7.12) | 669(6.52) | 0.179 |
| No, n(%) | 7381(93.66) | 2204(92.88) | 9585(93.48) |  |
| **Depression** |  |  |  |  |
| Yes, n(%) | 872(11.06) | 352(14.83) | 1224(11.94) | <0.001 |
| No, n(%) | 7009(88.94) | 2021(85.17) | 9030(88.06) |  |
| **CES-D-10, Mean(SD)** | 1.83(1.83) | 2.05(2.13) | 1.88(1.91) | <0.001 |
| **Smoking status** |  |  |  |  |
| Non-smoker, n(%) | 5572(70.70) | 1725(72.72) | 7297(71.17) | 0.028 |
| Former smoker, n(%) | 785(9.96) | 194(8.18) | 979(9.55) |  |
| Current smoker, n(%) | 1524(19.34) | 453(19.10) | 1977(19.28) |  |
| **Problematic drinking** |  |  |  |  |
| Yes, n(%) | 224(2.84) | 62(2.61) | 286(2.79) | 0.552 |
| No, n(%) | 7657(97.16) | 2311(97.39) | 9968(97.21) |  |
| **Regular exercise** |  |  |  |  |
| Yes, n(%) | 2983(37.85) | 949(39.99) | 3932(38.35) | 0.06 |
| No, n(%) | 4898(62.15) | 1424(60.01) | 6322(61.65) |  |

| **Supplementary Material 2. Sensitivity analysis: Hazard Ratios of Poor self-rated health between (Very Good, Good, Fair) and (Bad, Very Bad)** | | | | |
| --- | --- | --- | --- | --- |
|  |  | **Cognitively Intact**  **(n=6569)** | **Cognitively impaired**  **(n=1312)** | **Total**  **(n=7881)** |
| **Model 1** | **Self-rated health**  **(Ref : Very Good, Good, Fair)** | - | - | - |
|  | **Bad, Very Bad** | 1.81***(1.59 - 2.07) | 1.56***(1.26 - 1.92) | 1.84***(1.65 - 2.06) |
| **Model 2** | **Self-rated health**  **(Ref : Very Good, Good, Fair)** | - | - | - |
|  | **Bad, Very Bad** | 1.76***(1.54 - 2.01) | 1.57***(1.26 - 1.94) | 1.80***(1.61 - 2.01) |
| **Model 3** | **Self-rated health**  **(Ref : Very Good, Good, Fair)** | - | - | - |
|  | **Bad, Very Bad** | 1.52***(1.30 - 1.77) | 1.28*(1.01 - 1.63) | 1.51***(1.33 - 1.72) |
| **Model 4** | **Self-rated health**  **(Ref : Very Good, Good, Fair)** | - | - | - |
|  | **Bad, Very Bad** | 1.51***(1.29 - 1.76) | 1.33*(1.04 - 1.7) | 1.51***(1.33 - 1.71) |

| **Supplementary Material 3. Sensitivity analysis: Hazard Ratios of Poor self-rated health between (Very Good, Good) and (Fair, Bad, Very Bad)** | | | | |
| --- | --- | --- | --- | --- |
|  |  | **Cognitively Intact**  **(n=6569)** | **Cognitively impaired**  **(n=1312)** | **Total**  **(n=7881)** |
| **Model 1** | **Self-rated health**  **(Ref : Very Good, Good)** | - | - | - |
|  | **Fair, Bad, Very Bad** | 1.65***(1.40 - 1.93) | 1.15(0.84 - 1.56) | 1.62***(1.40 - 1.86) |
| **Model 2** | **Self-rated health**  **(Ref : Very Good, Good)** | - | - | - |
|  | **Fair, Bad, Very Bad** | 1.63***(1.39 - 1.92) | 1.15(0.84 - 1.57) | 1.59***(1.38 - 1.83) |
| **Model 3** | **Self-rated health**  **(Ref : Very Good, Good)** | - | - | - |
|  | **Fair, Bad, Very Bad** | 1.42***(1.20 - 1.68) | 0.93(0.67 - 1.29) | 1.36***(1.17 - 1.58) |
| **Model 4** | **Self-rated health**  **(Ref : Very Good, Good)** | - | - | - |
|  | **Fair, Bad, Very Bad** | 1.41***(1.19 - 1.68) | 0.93(0.67 - 1.29) | 1.34***(1.16 - 1.56) |

| **Supplementary Material 4. Sensitivity analysis: Hazard Ratios of Poor self-rated health using a different cutoff point(1SD)** | | | | |
| --- | --- | --- | --- | --- |
|  |  | **Cognitively Intact**  **(n=5986)** | **Cognitively impaired**  **(n=1895)** | **Total**  **(n=7881)** |
| **Model 1** | **Self-rated health**  **(Ref : Very Good)** | - | - | - |
|  | **Good** | 1.25(0.73 - 2.16) | 1.24(0.5 - 3.11) | 1.23(0.77 - 1.96) |
|  | **Fair** | 1.56(0.91 - 2.67) | 1.18(0.48 - 2.89) | 1.46(0.92 - 2.32) |
|  | **Bad** | 2.36**(1.37 - 4.05) | 1.71(0.71 - 4.15) | 2.25**(1.42 - 3.56) |
|  | **Very Bad** | 3.26***(1.83 - 5.78) | 2.85*(1.16 - 6.96) | 3.58***(2.23 - 5.75) |
| **Model 2** | **Self-rated health**  **(Ref : Very Good)** | - | - | - |
|  | **Good** | 1.26(0.73 - 2.16) | 1.29(0.52 - 3.24) | 1.24(0.78 - 1.98) |
|  | **Fair** | 1.57(0.91 - 2.7) | 1.23(0.50 - 3.03) | 1.48(0.93 - 2.35) |
|  | **Bad** | 2.32**(1.35 - 4) | 1.79(0.74 - 4.36) | 2.24**(1.41 - 3.56) |
|  | **Very Bad** | 3.02***(1.7 - 5.39) | 2.97*(1.21 - 7.29) | 3.46***(2.15 - 5.57) |
| **Model 3** | **Self-rated health**  **(Ref : Very Good)** | - | - | - |
|  | **Good** | 1.24(0.72 - 2.14) | 1.25(0.5 - 3.12) | 1.22(0.77 - 1.95) |
|  | **Fair** | 1.50(0.87 - 2.58) | 1.16(0.47 - 2.85) | 1.42(0.89 - 2.25) |
|  | **Bad** | 2.05*(1.18 - 3.55) | 1.56(0.64 - 3.82) | 1.98**(1.24 - 3.16) |
|  | **Very Bad** | 2.32**(1.27 - 4.24) | 2.16(0.86 - 5.41) | 2.53***(1.54 - 4.15) |
| **Model 4** | **Self-rated health**  **(Ref : Very Good)** | - | - | - |
|  | **Good** | 1.22(0.71 - 2.1) | 1.23(0.49 - 3.08) | 1.21(0.76 - 1.93) |
|  | **Fair** | 1.47(0.85 - 2.52) | 1.12(0.45 - 2.76) | 1.39(0.87 - 2.21) |
|  | **Bad** | 2.01*(1.16 - 3.48) | 1.53(0.62 - 3.76) | 1.94**(1.22 - 3.10) |
|  | **Very Bad** | 2.27**(1.24 - 4.15) | 2.02(0.80 - 5.08) | 2.41**(1.47 - 3.95) |

| **Supplementary Material 5.Sensitivity analysis: Hazard Ratios of Poor self-rated health using a different cutoff point(2SD)** | | | | |
| --- | --- | --- | --- | --- |
|  |  | **Cognitively Intact**  **(n=7047)** | **Cognitively impaired**  **(n=834)** | **Total**  **(n=7881)** |
| **Model 1** | **Self-rated health**  **(Ref : Very Good)** | - | - | - |
|  | **Good** | 1.26(0.75 - 2.14) | 1.67(0.58 - 4.78) | 1.23(0.77 - 1.96) |
|  | **Fair** | 1.58(0.94 - 2.65) | 1.22(0.44 - 3.38) | 1.46(0.92 - 2.32) |
|  | **Bad** | 2.40**(1.43 - 4.04) | 1.77(0.65 - 4.79) | 2.25**(1.42 - 3.56) |
|  | **Very Bad** | 3.50***(2.04 - 6.01) | 2.98*(1.09 - 8.15) | 3.58***(2.23 - 5.75) |
| **Model 2** | **Self-rated health**  **(Ref : Very Good)** | - | - | - |
|  | **Good** | 1.26(0.74 - 2.12) | 1.78(0.62 - 5.14) | 1.24(0.78 - 1.98) |
|  | **Fair** | 1.58(0.94 - 2.65) | 1.29(0.46 - 3.60) | 1.48(0.93 - 2.35) |
|  | **Bad** | 2.35**(1.39 - 3.95) | 1.90(0.69 - 5.21) | 2.24**(1.41 - 3.56) |
|  | **Very Bad** | 3.28***(1.91 - 5.65) | 3.22*(1.17 - 8.85) | 3.46***(2.15 - 5.57) |
| **Model 3** | **Self-rated health**  **(Ref : Very Good)** | - | - | - |
|  | **Good** | 1.24(0.74 - 2.10) | 1.67(0.58 - 4.83) | 1.22(0.77 - 1.95) |
|  | **Fair** | 1.51(0.9 - 2.55) | 1.19(0.43 - 3.34) | 1.42(0.89 - 2.25) |
|  | **Bad** | 2.09**(1.23 - 3.54) | 1.61(0.58 - 4.46) | 1.98**(1.24 - 3.16) |
|  | **Very Bad** | 2.51**(1.43 - 4.40) | 2.28(0.79 - 6.57) | 2.53***(1.54 - 4.15) |
| **Model 4** | **Self-rated health**  **(Ref : Very Good)** | - | - | - |
|  | **Good** | 1.22(0.72 - 2.06) | 1.62(0.56 - 4.71) | 1.21(0.76 - 1.93) |
|  | **Fair** | 1.48(0.88 - 2.50) | 1.14(0.40 - 3.21) | 1.39(0.87 - 2.21) |
|  | **Bad** | 2.04**(1.21 - 3.46) | 1.63(0.58 - 4.54) | 1.94**(1.22 - 3.10) |
|  | **Very Bad** | 2.39**(1.36 - 4.20) | 2.19(0.76 - 6.32) | 2.41**(1.47 - 3.95) |
